# Supplementary material for: Risk Assessment of Perioperative Respiratory Adverse Events and Validation of the COLDS Score in Children with Upper Respiratory Tract Infection
Source: Medicina (Kaunas). 2022 Sep 23;58(10):1340. doi: 10.3390/medicina58101340 (PMC9611335; doi:10.3390/medicina58101340)
Supplement: Supplementary file 1 [file medicina-58-01340-s001.zip › Proofread_medicina-1868449-supplementary_revised.pdf]

1 Supplementary materials

Supplementary Table S1. The ‘COLDS’ score table.

	Score point		
	1	2	5
C (current signs and symptoms)	None	Mild	Moderate/Severe
O (onset of symptoms)	> 4 weeks ago	2-4 weeks ago	< 2 weeks ago
L (presence of lung disease)	None	Mild	Moderate/Severe
D (airway device)	None or Facemask	LMA or supraglottic airway	Endotracheal tube
S (type of surgery)	Other	Minor airway	Major airway

LMA: laryngeal mask airway. From Lee BJ, August DA. COLDS: A heuristic preanesthetic risk score for children with upper respiratory tract infection. Paediatr Anaesth. (2014) 24:349-50.

Supplementary Table S2. Initial logistic regression model for perioperative respiratory adverse events (PRAEs) prediction.

Independent variables	OR	95% CI of OR	P-value
(constant)	0.033		0.003
Age (years)	0.828	0.688-0.997	0.046
Sex (male)	1.156	0.465-2.877	0.755
Weight (kg)	1.010	0.965-1.056	0.673
Anesthetic time (min)	1.000	0.996-1.005	0.830
'COLDS' score	1.224	1.022-1.466	0.028
Ongoing URTI symptom	2.880	1.095-7.576	0.032

Adjusted $R^2 = 0.224$, Hosmer & Lemeshow test P=0.139, classification accuracy 80.4%. (OR: odd ratio, CI: confidential interval, URTI: upper respiratory tract infection).

Supplementary Table S3. Demographic information of low COLDS group (Group L) and high COLDS group (Group H) before matching.

Group	Group L (N = 89)	Group H (N = 69)	P value
Sex (M/F)	58/31	43/26	0.711
Age (yr)	6.01 ± 4.85	7.96 ± 5.24	0.018
Height (cm)	109.05 ± 28.04	123.32 ± 32.91	0.006
Weight (kg)	24.02 ± 22.24	30.07 ± 18.88	0.072
Cough (Y/N)	47/33	47/20	0.152
Sputum (Y/N)	15/64	24/45	0.030
Rhinorrhea (Y/N)	48/33	27/40	0.022
Fever (Y/N)	21/58	18/49	0.969
Preoperative abnormal active lesion on x- ray (Y/N)	0/89	8/61	0.001
Duration of URTI symptom (day)	11.59 ± 11.06	9.06 ± 13.50	0.236
Active URTI (Y/N)	16/73	36/33	<0.001
URTI onset			
> 1 month before surgery	8	0	<0.001
1 month~1 week before surgery	53	15	
1 day~1 week before surgery	12	18	
Within 1 day before surgery	16	36	

Type or surgery (major/minor)	18/71	21/48	0.140
Department of surgery (CS/GS/OS/PS/OL/OP/Others)	1/6/22/6/21/5/28	4/11/15/4/14/4/17	0.362
Anesthetic time (min)	120.61 ± 114.28	126.38 ± 93.24	0.734
Operation time (min)	69.11 ± 78.77	85.17 ± 83.13	0.217
Duration at PACU (min)	59.65 ± 12.12	62.58 ± 10.12	0.116
C	1.20 ± 0.40	1.68 ± 0.96	<0.001
O	1.85 ± 0.73	4.43 ± 1.25	<0.001
L	1.03 ± 0.18	1.68 ± 1.47	0.001
D	4.58 ± 1.19	5 ± 0	0.001
S	1.07 ± 0.25	1.45 ± 1.14	0.008
Total COLDS score	9.73 ± 1.38	14.25 ± 1.55	<0.001

URTI: upper respiratory tract infection, CS: cardiac surgery, GS: general surgery, OS: orthopedic surgery, PS: plastic surgery, OL: otorhinolaryngology, OP: ophthalmology, PACU: post anesthesia care unit, C: current symptoms, O: onset of URTI symptoms, L: lung disease, D: airway device, S: surgery type.
